# Establishing a predictive model for liver fluke infection on the basis of early changes in laboratory indicators: a retrospective study

**DOI:** 10.1186/s13071-025-06833-9

**Published:** 2025-05-22

**Authors:** Yiting Wang, Tie Wang, Xin Wen, Chongchong Feng

**Affiliations:** 1 Department of Laboratory Medicine, Second Hospital of Jilin University, Changchun, China; 2 Department of Laboratory Medicine, First Hospital of Jilin University, Changchun, China

**Keywords:** Liver fluke, Prediction model, Laboratory indicators, Nomogram, Logistic regression

## Abstract

**Background:**

Hepatic clonorchiasis is one of the most prevalent foodborne parasitic diseases in China and is often overlooked because the initial symptoms are not obvious. In this study, a multivariate model for the early prediction of disease onset using laboratory test data from liver-fluke-infected patients was developed and validated.

**Methods:**

Laboratory data from 147 liver-fluke-infected patients and 151 healthy control subjects were collected. Univariate logistic regression, Spearman correlation analysis, and collinearity diagnosis were used to screen for independent factors. A multivariate model was then constructed using the backward likelihood ratio method. For external validation, an independent patient cohort from another hospital was analyzed. The discriminative performance of the combined model was compared with that of previously identified biomarkers (eosinophil count and γ-glutamyl transpeptidase).

**Results:**

A 12-indicator prediction model for liver fluke infection was developed using traditional logistic regression (82.31% sensitivity and 88.08% specificity). The receiver operating characteristic curve, calibration curve, and decision curve analyses revealed that the model exhibited excellent discriminative ability (area under the curve [AUC]: training = 0.928, validation = 0.808), goodness of fit, and clinical practicability. The combined model showed superior discrimination compared with individual biomarkers, including eosinophil count (AUC = 0.577) and γ-glutamyl transpeptidase (AUC = 0.620).

**Conclusions:**

This study developed an early risk prediction model for liver fluke infection using routine laboratory test data. Compared with previously reported biomarkers, the model demonstrated superior diagnostic performance and showed potential as a clinical tool for identifying early stage liver fluke infection in patients.

**Graphical Abstract:**

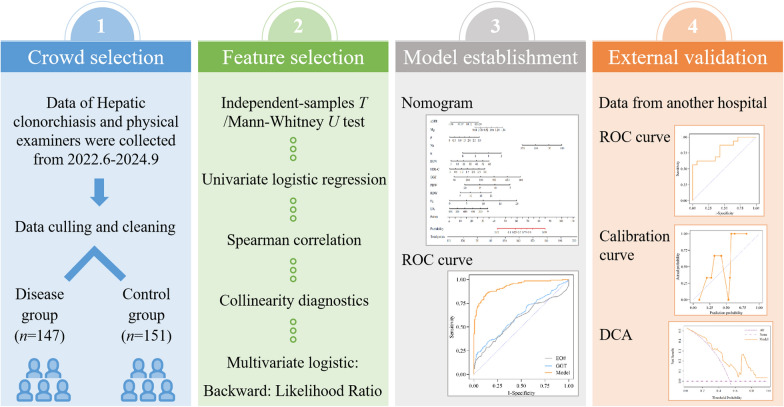

## Background

Hepatic clonorchiasis, caused by the ingestion of raw or undercooked freshwater fish and shrimp containing infectious larvae, represents the most prevalent food-borne parasitic disease in China. Following consumption, the larvae migrate to the hepatobiliary system, where they mature into adult flukes that chronically parasitize the bile ducts [1]. *Clonorchis sinensis*, *Opisthorchis viverrini*, and *Opisthorchis felineus* are three liver fluke species that pose significant threats to human health [2]. Among them, *C. sinensis* remains the most prevalent in Asia, with approximately 15 million current infections and an estimated 200 million people at risk of chronic infection [3]. Liver fluke infection shows no significant predilection for age, sex, or nationality, thus all populations are susceptible to it. Hepatic clonorchiasis typically follows a chronic course, with initial clinical manifestations often being subtle, primarily nonspecific symptoms such as dyspepsia, epigastric discomfort, fatigue, and mild depression. This insidious presentation frequently leads to delayed diagnosis and treatment [4]. Adult liver flukes exhibit remarkable longevity in human hosts, persisting for 20–30 years. This chronic infection induces cumulative hepatobiliary damage. Severe cases manifest as cholecystitis, cholangitis, and cholelithiasis, with potential progression to cirrhosis, hepatocellular carcinoma, cholangiocarcinoma, and ultimately, multiorgan failure with high mortality [5, 6]. The World Health Organization International Agency for Research on Cancer has designated liver flukes as group I biological carcinogens for cholangiocarcinoma [7]. Drug deworming has a good effect on early liver fluke infection. Therefore, early diagnosis, monitoring, and intervention of liver fluke infection are very important.

At present, the gold standard for the clinical diagnosis of liver fluke disease is the detection of liver fluke eggs through routine stool examination, supplemented by immunological tests, molecular diagnosis, ultrasound, imaging, and other examinations. However, some tests have inherent delays and are difficult to perform effectively during early stage infection [8, 9]. Fecal examination results depend heavily on the examiner’s professional skill and attention to detail during microscopy. Moreover, the detection rate of single stool sample microscopy for parasite eggs remains suboptimal, frequently leading to missed diagnosis and delayed treatment. Blind microscopy is time consuming and impractical for routine use. Immunological antibody testing is prone to misdiagnosis because of its high false-positive rate and strong dependence on temperature and time variations. Molecular diagnostic methods remain cost-prohibitive and vulnerable to sample contamination issues. Previous studies have focused predominantly on imaging examinations and clinical symptom analysis in infected patients, which are approaches that incur additional healthcare costs and impose physical or economic burdens on patients. Therefore, the development of a convenient, rapid, efficient, and easily implemented early diagnostic method is considered clinically essential for increasing therapeutic efficacy and improving prognostic outcomes in hepatic clonorchiasis management.

This retrospective study was conducted in Jilin Province, China, to identify early infection indicators and establish a predictive model for hepatic clonorchiasis.

## Methods

### Study design and participants

Patient data *(n* = 147) with confirmed liver fluke infections were extracted from the electronic medical records system of the Second Hospital of Jilin University from July 2022 to September 2024. The collected parameters included sex, age, comorbidities, and initial laboratory results upon admission. Health control data were derived from contemporaneous physical examination center data. The above data were used as a training set to construct a predictive model. The diagnostic criterion for hepatic clonorchiasis was detection of hepatic flukes via routine microscopic examination of the stool. The inclusion and exclusion criteria for physical examination center data were as follows: (1) eliminate data that was seriously missing information; (2) disqualified samples, such as those associated with hemolysis, lipemia, and jaundice, were excluded; and (3) medical and laboratory tests: data were excluded according to the screening criteria in WS/T 645.2-2018 [10]. External validation employed an independent cohort from the First Hospital of Jilin University (July–October 2024; 16 cases and 14 controls), with identical inclusion/exclusion protocols.

Previous studies have identified γ-glutamyl transpeptidase (GGT) and the eosinophil count (EO#) as potential diagnostic biomarkers for liver fluke infection [11–13]. EO# is particularly sensitive to parasitic infections, as eosinophils undergo degranulation and release cytotoxic proteins upon parasite contact. On the basis of this evidence, we conducted a comparative performance analysis between these established biomarkers and our novel prediction model.

### Data collection

The laboratory tests included hematological and biochemical tests. The blood and biochemical equipment used at the Second Hospital of Jilin University were BC-6800 (Mindray Biomedical Electronics Corp, Shenzhen, China), XN-2000 (Sysmex Corp, Hyogo, Japan), CS-5100 (Sysmex Corp, Hyogo, Japan), HISC-800 (Sysmex Corp, Hyogo, Japan), and 008AS (Hitachi High-Technologies, Tokyo, Japan). The blood and biochemical equipment used at the First Hospital of Jilin University were XN-9000 (Sysmex Corp, Hyogo, Japan), CS-5100 (Sysmex Corp, Hyogo, Japan), and 7600-210 (Hitachi High-Technologies, Tokyo, Japan). This study was approved by the Ethics Committee of the Second Hospital of Jilin University (no. 2025134). The requirement for written informed consent was waived owing to the retrospective nature of the study.

### Data cleaning

Some patient laboratory data were missing because of the absence of certain types of test results. To ensure the accuracy, consistency, and applicability of the data, the original data collected were eliminated, and missing values were processed: (1) data elimination: original data were summarized according to time, date, test items, etc., and the data with complete duplication (the same patient) were eliminated and (2) processing of missing data values: to avoid missing important information, variables with a missing rate greater than 30% were eliminated according to the processing of missing values. For the remaining missing values, quantitative data were imputed using mean substitution (normally distributed) or median replacement (nonnormally distributed), whereas categorical data were randomly imputed proportional to observed category frequencies, ensuring methodological appropriateness for subsequent analyses.

### Statistical analysis

The Kolmogorov‒Smirnov test was used to assess the normality of the quantitative data. The quantitative data with a normal distribution were compared via an independent samples *t* test and are expressed as $\overline{x }\pm s$ (average ± standard deviation). The quantitative data with a nonnormal distribution were compared via the Mann‒Whitney *U* test and expressed as* M* (*P*_25_, *P*_75_) [median (25th percentile, 75th percentile)]. The independent factors of early liver fluke infection were screened through various methods. Variables showing significant differences between groups were analyzed by univariate logistic regression, and variables with *P* ≥ 0.05 were excluded. Spearman correlation analysis was used to determine whether there was a significant correlation between variables, and collinearity diagnostics were used to screen variables to avoid the influence of multicollinearity on model accuracy. In general, a variance inflation factor (VIF) > 5 and tolerance < 0.2 indicate that multicollinearity might exist among independent variables and should be eliminated. Finally, the required variables were included in the multivariate logistic analysis, and the backward likelihood ratio method was used to fit the multivariate model. The odds ratio (OR) and 95% confidence interval (*CI*) of each variable were calculated. A nomogram was generated from the combined model. The receiver operating characteristic (ROC) curve was used to evaluate the discrimination of the prediction model, and the area under the curve (AUC) and its 95% *CI* were calculated. An AUC of > 0.75 was considered to indicate good model performance. A* P* value of < 0.05 was considered statistically significant. A calibration curve was used to evaluate the model’s goodness of fit, and a *P* value of > 0.05 was considered a satisfactory fit. Decision curve analysis (DCA) was used to evaluate the clinical effectiveness of the model. Stata 15.0 (Stata Corp LLC, Texas, USA), GraphPad Prism 8.0 (GraphPad Software Corp, San Diego, CA, USA), and SPSS 23.0 (IBM Corp, Armonk, NY, USA) were used for data analysis and graphical plotting.

## Results

### Clinical and laboratory characteristics of patients with liver fluke infection upon admission

A total of 147 patients with liver fluke infection (disease group) and 151 healthy subjects (control group) were included in the study. The average age of the disease group was 57.89 years, of which 78.91% were male. The average age of the individuals in the control group was 55.28 years, and 77.48% of the individuals were male. In the disease group, the most common comorbidity was cardiovascular disease (41.50%), of which coronary atherosclerotic heart disease accounted for the highest proportion, and the second most common comorbidity was cerebrovascular disease (10.88%).

The above data were used as a training set to build the model. Kolmogorov‒Smirnov test results revealed that, with the exception of globulin (GLB), uric acid (UA), and low-density lipoprotein cholesterol (LDL-C), the other laboratory indices exhibited a skewed distribution (*P* < 0.05). The 60 conventional laboratory indicators had different degrees of missing data (0.67–15.10%), and the mean and median interpolation were used. *T* and *U* tests revealed that 39 indices were significantly different between the two groups (*P* < 0.05). The results of the first laboratory examination after admission are presented in Table 1.

**Table 1:** First laboratory test results after admission

Analytes	Disease group (*n* = 147)	Control group (*n* = 151)	*t* / *Z*-value	*P*-value
	*M* (*P*_25_, *P*_75_) /$\overline{x }\pm s$	*M* (*P*_25_, *P*_75_) /$\overline{x }\pm s$		
WBC, × 10^9^/L	7.10 (5.40, 8.30)	6.00 (5.20, 7.50)	−2.953	0.003
NE#, × 10^9^/L	4.37 (3.08, 5.95)	3.49 (2.78, 4.49)	−4.106	< 0.001
LY#, × 10^9^/L	1.60 (1.10, 2.10)	2.00 (1.70, 2.50)	−6.007	< 0.001
MO#, × 10^9^/L	0.40 (0.40, 0.60)	0.30 (0.30, 0.40)	−7.112	< 0.001
EO#, × 10^9^/L	0.15 (0.08, 0.30)	0.12 (0.07, 0.19)	−2.245	0.025
BA#, × 10^9^/L	0.03 (0.02, 0.05)	0.03 (0.02, 0.04)	−1.523	0.128
NE%, %	64.40 (56.05, 72.55)	58.00 (52.90, 62.40)	−5.214	< 0.001
LY%, %	23.20 (17.20, 31.45)	33.40 (29.40, 38.30)	−7.951	< 0.001
MO%, %	6.80 (5.50, 8.30)	5.30 (4.60, 6.30)	−6.421	< 0.001
EO%, %	2.50 (1.10, 4.00)	1.90 (1.30, 3.00)	−1.736	0.083
BA%, %	0.50 (0.30, 0.80)	0.50 (0.30, 0.70)	−0.842	0.400
RBC, × 10^12^/L	4.64 (4.12, 5.00)	4.95 (4.68, 5.26)	−6.061	< 0.001
HGB, g/L	143.00 (122.50, 156.00)	157.00 (147.00, 166.00)	−6.711	< 0.001
HCT, %	42.20 (37.15, 46.10)	46.60 (43.80, 48.70)	−6.983	< 0.001
MCV, fL	92.60 (88.70, 96.10)	93.10 (90.30, 95.80)	−1.295	0.195
MCH, pg	31.10 (29.60, 32.45)	31.20 (30.50, 32.30)	−1.139	0.255
MCHC, g/L	336.00 (329.00, 343.00)	337.00 (332.00, 341.00)	−0.714	0.475
RDW, %	13.10 (12.60, 13.70)	12.80 (12.60, 13.20)	−3.424	0.001
PLT, × 10^9^/L	215.00 (176.30, 275.00)	230.00 (196.00, 264.00)	−1.797	0.072
PCT, %	0.22 (0.18, 0.27)	0.23 (0.20, 0.26)	−1.032	0.302
MPV, fL	10.10 (9.20, 10.90)	9.80 (9.10, 10.40)	−1.982	0.047
PDW, %	16.00 (13.10, 16.40)	16.20 (15.90, 16.40)	−3.453	0.001
APTT, s	31.50 (29.50, 33.70)	32.10 (29.90, 34.00)	−0.744	0.457
APTT-ratio	1.03 (0.96, 1.10)	1.03 (0.96, 1.10)	−0.514	0.607
PT, s	11.30 (10.60, 11.90)	10.60 (10.30, 11.00)	−5.817	< 0.001
PTA, %	100.00 (90.00, 111.00)	106.00 (100.00, 113.00)	−3.774	< 0.001
TT, s	13.40 (12.80, 14.60)	13.60 (12.90, 14.30)	−0.287	0.774
INR	1.00 (0.94, 1.08)	0.97 (0.94, 1.01)	−3.249	0.001
Fg, g/L	3.35 (2.82, 4.26)	2.88 (2.55, 3.26)	−5.374	< 0.001
GLU, mmol/L	5.70 (4.97, 6.82)	5.37 (5.03, 5.87)	−2.182	0.029
Cr, μmol/L	74.00 (63.00, 89.75)	74.00 (66.00, 82.00)	−0.734	0.463
UA, mmol/L	328.78 $\pm$ 111.98	379.44 $\pm$ 92.36	−4.248	< 0.001
BUN, mmol/L	6.13 (4.65, 7.98)	5.36 (4.62, 6.48)	−2.317	0.021
eGFR, ml/min	94.90 (74.90, 103.90)	102.60 (91.10, 108.50)	−4.550	< 0.001
TP, g/L	67.30 (61.60, 73.33)	74.50 (72.60, 77.40)	−9.235	< 0.001
ALB, g/L	41.10 (36.80, 44.10)	46.30 (45.00, 47.70)	−10.943	< 0.001
GLB, g/L	27.04 $\pm$ 5.30	28.56 $\pm$ 3.27	−2.933	0.004
A/G	1.60 (1.30, 1.80)	1.64 (1.48, 1.76)	−3.218	0.001
AST, U/L	19.00 (15.00, 28.00)	23.00 (20.00, 27.00)	−3.818	< 0.001
ALT, U/L	23.00 (15.00, 32.75)	26.00 (19.00, 32.00)	−2.189	0.029
ALP, U/L	82.00 (67.00, 102.75)	75.00 (64.00, 92.00)	−2.464	0.014
GGT, U/L	41.00 (24.25, 79.75)	31.00 (21.00, 48.00)	−3.592	< 0.001
TBIL, μmol/L	12.03 (8.56, 17.18)	12.81 (9.95, 16.44)	−0.766	0.444
DBIL, μmol/L	3.66 (2.34, 5.34)	3.35 (2.65, 4.38)	−1.615	0.106
IBIL, μmol/L	8.49 (5.78, 11.83)	9.34 (7.16, 11.72)	−1.888	0.059
CK, U/L	67.00 (48.00, 99.50)	101.00 (78.00, 140.00)	−6.234	< 0.001
LDH, U/L	193.00 (165.50, 238.00)	192.00 (176.00, 204.00)	−0.627	0.531
TG, mmol/L	1.60 (1.12, 2.43)	1.73 (1.18, 2.80)	−0.939	0.348
TC, mmol/L	4.65 (3.55, 5.48)	4.90 (4.34, 5.48)	−2.485	0.013
HDL-C, mmol/L	1.04 (0.84, 1.24)	1.19 (0.98, 1.43)	−4.494	< 0.001
LDL-C, mmol/L	2.79 $\pm$ 1.06	2.93 $\pm$ 0.79	−1.186	0.237
Na, mmol/L	141.80 (139.40, 143.75)	142.30 (141.00, 144.00)	−2.673	0.008
K, mmol/L	3.98 (3.76, 4.19)	4.20 (3.99, 4.37)	−5.130	< 0.001
Cl, mmol/L	105.00 (102.35, 108.00)	105.00 (104.00, 107.00)	−0.093	0.926
Ca, mmol/L	2.23 (2.15, 2.29)	2.29 (2.23, 2.35)	−5.080	< 0.001
P, mmol/L	1.08 (0.96, 1.25)	1.02 (0.92, 1.12)	−3.260	0.001
Mg, mmol/L	0.90 (0.84, 0.97)	0.95 (0.90, 0.99)	−4.156	< 0.001
FT_3_, pmol/L	4.79 (4.14, 5.46)	5.28 (4.82, 5.69)	−4.401	< 0.001
FT_4_, pmol/L	16.30 (14.50, 18.00)	16.00 (14.70, 17.20)	−0.905	0.366
TSH, mIu/L	1.76 (1.14, 2.73)	1.79 (1.16, 2.95)	−0.155	0.877

WBC, white blood cell; NE#, neutrophil count; LY#, lymphocyte count; MO#, monocyte count; EO#, eosinophil count; BA#, basophil count; NE%, neutrophil percentage; LY%, lymphocyte percentage; MO%, monocyte percentage; EO%, eosinophil percentage; BA%, basophil percentage; RBC, red blood cell; HGB, hemoglobin; HCT, hematocrit; MCV, mean corpuscular volume; MCH, mean corpuscular hemoglobin; MCHC, mean corpuscular hemoglobin concentration; RDW, red blood cell distribution width; PLT, platelet count; PCT, plateletcrit; MPV, mean platelet volume; PDW, platelet distribution width; APTT, activated partial thromboplastin time; PT, prothrombin time; PTA, prothrombin time activity; TT, thrombin time; INR, international normalized ratio; Fg, fibrinogen; GLU, glucose; Cr, creatinine; UA, uric acid; BUN, blood urea nitrogen; eGFR, estimated glomerular filtration rate; TP, total protein; ALB, albumin; GLB, globulin; A/G, albumin–globulin ratio; AST, aspartate aminotransferase; ALT, alanine aminotransferase; ALP, alkaline phosphatase; GGT, γ-glutamyl transpeptidase; TBIL, total bilirubin; DBIL, direct bilirubin; IBIL, indirect bilirubin; CK, creatine kinase; LDH, lactate dehydrogenase; TG, triglyceride; TC, total cholesterol; HDL-C, high-density lipoprotein cholesterol; LDL-C, low-density lipoprotein cholesterol; Na, sodium; K, potassium; Cl, chloride; Ca, calcium; P, phosphorus; Mg, magnesium; FT_3_, free triiodothyronine; FT_4_, free tetraiodothyronine; TSH, thyrotropin; $\overline{x }\pm s$, Average ± standard deviation; *M* (*P*_25_, *P*_75_), median (25th percentile, 75th percentile)

### Feature selection and model establishment

**Figure 1:**
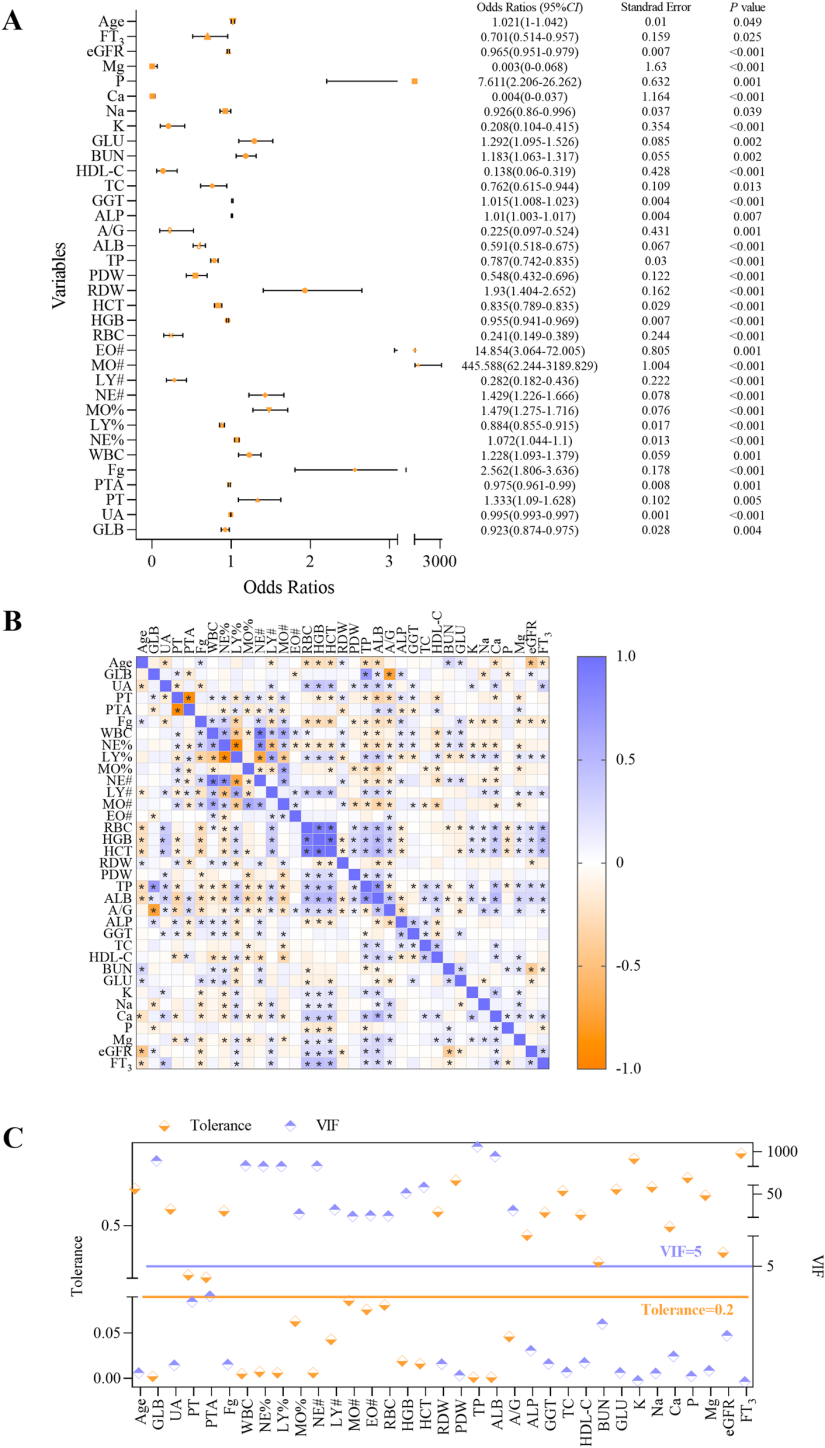
Process of feature selection. **A** Forest plot based on univariate logistic regression analysis. **B** Correlation heat plot of 35 significant differences. **C** Collinearity diagnostics. In Fig. 1C, the left axis represents tolerance, with data points and lines shown in yellow; the right axis represents VIF, with data points and lines shown in purple. *CI*, confidence interval; VIF, variance inflation factor. *: *P* < 0.05.

**Figure 2:**
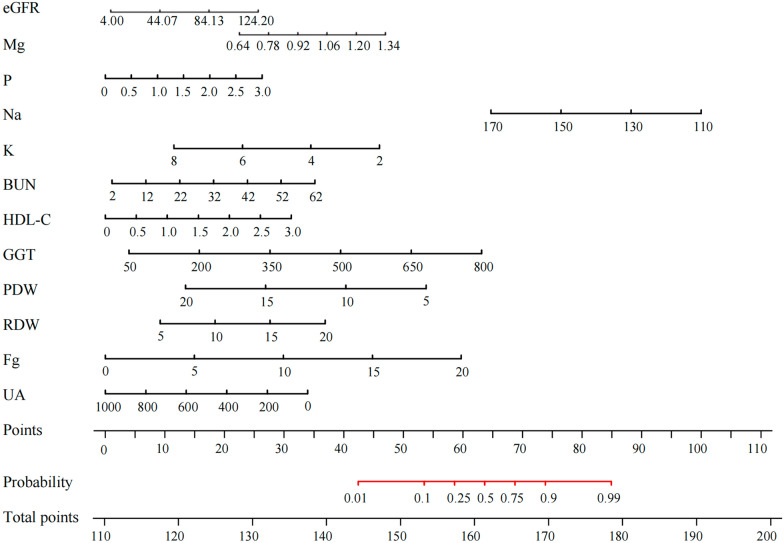
Nomogram

Univariate logistic analysis revealed that 35 variables were independent risk factors for liver fluke infection: age, GLB, UA, prothrombin time (PT), prothrombin time activity (PTA), fibrinogen (Fg), white blood cell (WBC), neutrophil percentage (NE%), lymphocyte percentage (LY%), monocyte percentage (MO%), neutrophil count (NE#), lymphocyte count (LY#), monocyte count (MO#), EO#, red blood cell (RBC), hemoglobin (HGB), hematocrit (HCT), red blood cell distribution width (RDW), platelet distribution width (PDW), total protein (TP), albumin (ALB), albumin‒globulin ratio (A/G), alkaline phosphatase (ALP), GGT, total cholesterol (TC), high-density lipoprotein cholesterol (HDL-C), blood urea nitrogen (BUN), glucose (GLU), potassium (K), sodium (Na), calcium (Ca), phosphorus (P), magnesium (Mg), estimated glomerular filtration rate (eGFR), and free triiodothyronine (FT_3_) (*P* < 0.05) (Fig. 1A). Spearman correlation analysis was performed on the above variables, and the results revealed significant correlations between most of them (*P* < 0.05) (Fig. 1B). To eliminate redundant indicators and avoid covariance among highly correlated indicators, we performed collinearity diagnostics to screen the variables for subsequent inclusion in the multifactor model. The results revealed that GLB, WBC, NE%, LY%, MO%, NE#, LY#, MO#, EO#, RBC, HGB, HCT, TP, ALB, and A/G had multicollinearity (VIF > 5 and tolerance < 0.2) (Fig. 1C), thus these 15 variables were excluded from subsequent calculations. The remaining indicators were incorporated into multifactor logistic regression, and the backward likelihood ratio method was fitted to the model. A model with 12 indicators, including UA, Fg, RDW, PDW, GGT, HDL-C, BUN, K, Na, P, Mg, and eGFR, was generated to predict early liver fluke infection in patients (Table 2). A nomogram was generated from the combined model, each variable was assigned a score, and the total score was calculated by summing the individual scores to reflect the probability of the patient’s disease (Fig. 2).

**Table 2:** Characteristics of the combined model

	*B*	Standard error	Wald	*P*	OR	95%*CI* of OR	
						Lower limit	Upper limit
UA	−0.009	0.002	17.174	< 0.001	0.991	0.987	0.995
Fg	0.802	0.293	7.515	0.006	2.231	1.257	3.958
RDW	0.495	0.266	3.473	0.062	1.641	0.975	2.762
PDW	−0.723	0.176	16.833	< 0.001	0.485	0.344	0.686
GGT	0.021	0.006	12.885	< 0.001	1.021	1.01	1.033
HDL-C	−2.796	0.697	16.079	< 0.001	0.061	0.016	0.24
BUN	0.152	0.105	2.108	0.146	1.165	0.948	1.431
K	−1.544	0.535	8.341	0.004	0.213	0.075	0.609
Na	−0.158	0.075	4.451	0.035	0.854	0.737	0.989
P	2.355	0.992	5.634	0.018	10.539	1.507	73.681
Mg	−9.436	2.996	9.917	0.002	0	0	0.028
eGFR	−0.055	0.015	14.573	< 0.001	0.946	0.919	0.973

*B*, regression coefficient; OR, odds ratio; CI, confidence interval

### Evaluation, comparison, and external validation of the models

**Figure 3:**
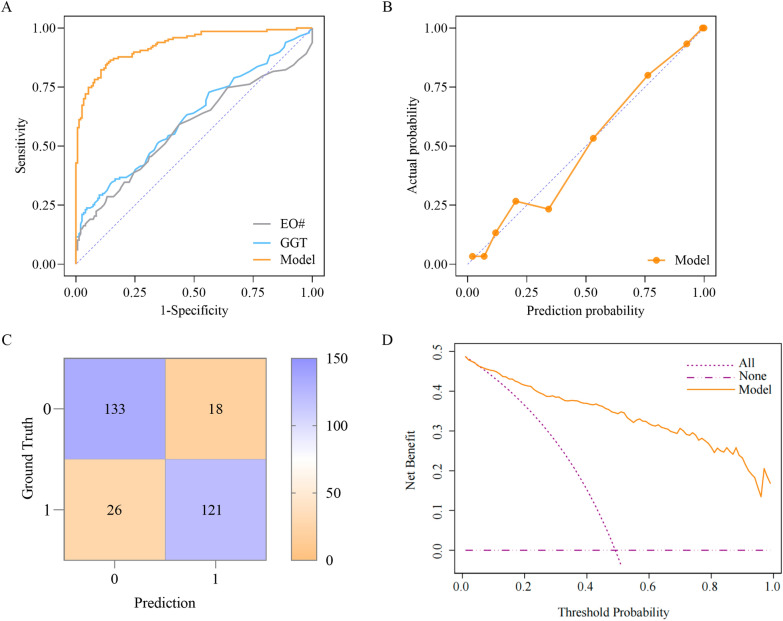
Performance evaluation of the combined model (training set). **A** ROC curve. **B** Calibration curve. **C** Confusion matrix: 0 for negative and 1 for positive. **D** DCA curve

**Figure 4:**
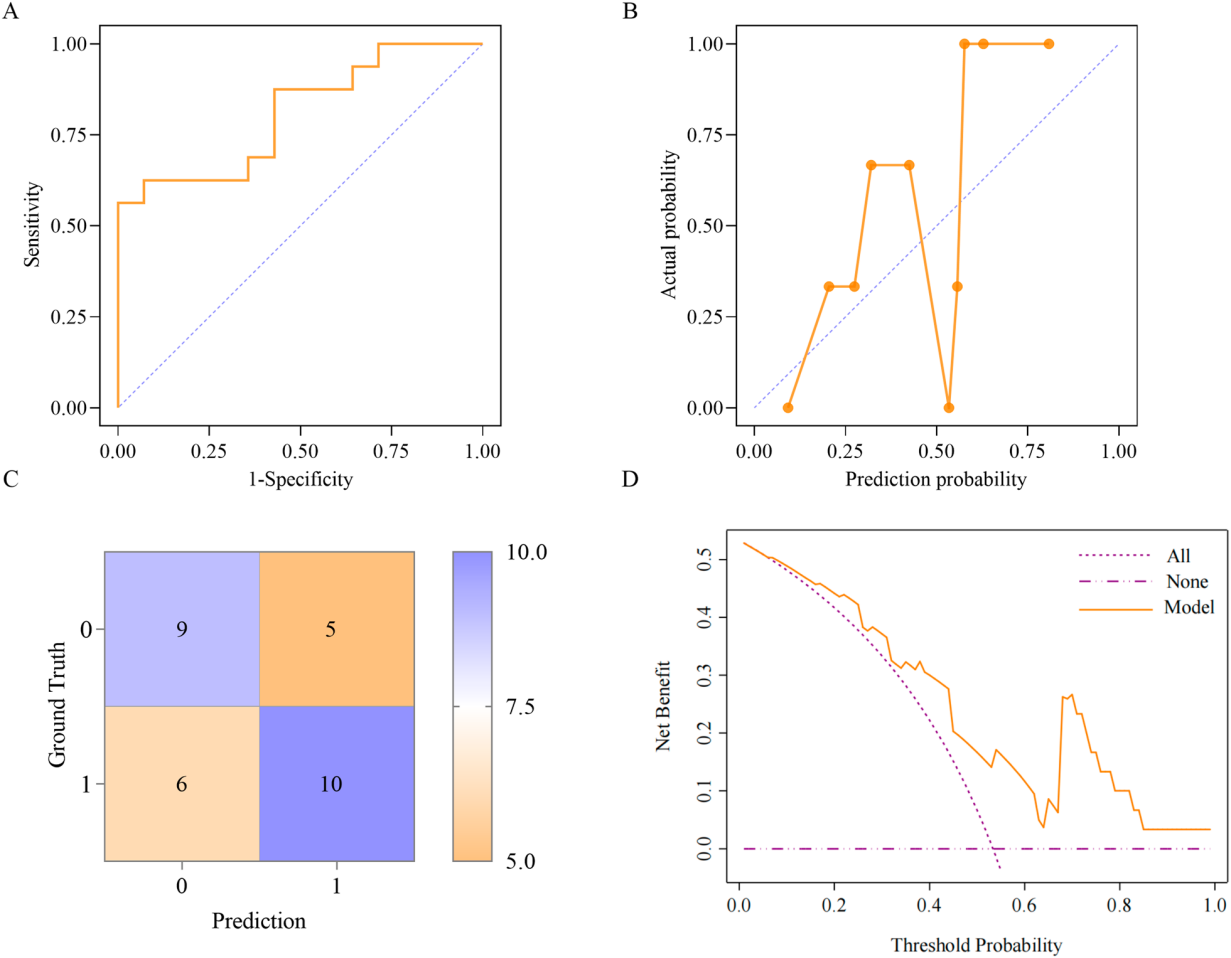
Performance evaluation of the combined model (validation set). **A** ROC curve. **B** Calibration curve. **C** Confusion matrix: 0 for negative and 1 for positive. **D** DCA curve

The ROC curve revealed that the combined model had good discrimination ability (AUC = 0.928, 95% CI 0.899–0.957, *P* < 0.001) (Fig. 3A). Compared with the combined model, EO# and GGT showed lower discrimination (EO#: AUC = 0.577, 95% CI 0.511–0.642; GGT: AUC = 0.620, 95% CI 0.557–0.683). External validation also confirmed good discrimination in the prediction model (AUC = 0.808, 95% CI 0.653–0.963, *P* = 0.004) (Fig. 4A). The Hosmer‒Lemeshow goodness-of-fit test of the calibration curve indicated excellent fit for the combined model (training set: *χ*^2^ = 3.735, *P* = 0.880 > 0.05; validation set: *χ*^2^ = 11.786, *P* = 0.162 > 0.05) (Fig. 3B, Fig. 4B). The confusion matrices were used to compare the model predictions with the actual outcomes (Fig. 3C, Fig. 4C). In the training set (*n* = 298), the results were as follows: true-positive (TP) = 121, true-negative (TN) = 133, false-positive (FP) = 18, and false-negative (FN) = 26. The calculated metrics were as follows: sensitivity = TP/(TP + FN) = 82.31%, specificity = TN/(TN + FP) = 88.08%, positive predictive value = TP/(TP + FP) = 87.05%, negative predictive value = TN/(TN + FN) = 83.65%, and overall accuracy = (TP + TN)/total sample = 85.23%. In the verification set, the model performance metrics were as follows: sensitivity = 62.5% [10/(10 + 6)], specificity = 64.29% [9/(9 + 5)], and overall accuracy = 63.33% [(10 + 9)/30]. DCA revealed a clear net benefit for the combined model (Fig. 3D, Fig. 4D). In the training set, when the threshold probability exceeded 8%, the model’s net benefit surpassed that of the “Treat All” strategy, with an increasing advantage at higher thresholds. The model consistently outperformed the “Treat None” strategy across all threshold probabilities. Above the 49.3% threshold probability, while extreme strategies showed zero or negative net benefits, the combined model maintained significant net benefits. In the validation set, when the threshold probability was in the range of 67–84%, the net benefit of the prediction model was apparently greater than that of both extreme strategies (“Treat All” and “Treat None”). When the threshold probability was within other ranges, the model’s net benefit was only slightly greater than those of these two strategies.

## Discussion

*Clonorchis sinensis* is an important food-borne liver fluke distributed throughout several Asian countries, particularly China [14]. Because early stage infections typically lack obvious symptoms, cases are frequently misdiagnosed or missed [15]. Therefore, in this study, a rapid and cost-effective predictive method based on laboratory indicators for the early detection of liver fluke infection was developed.

This study had the following innovations: (1) we established a nomogram prediction model on the basis of routine laboratory data and demonstrated that this rapid, noninvasive, and inexpensive method could be used for early screening of liver fluke infection; (2) during model construction, we employed multiple statistical methods, including the Mann‒Whitney *U* test, independent samples *t* test, univariate logistic regression, Spearman correlation analysis, collinearity diagnostics, and the backward likelihood ratio method, to jointly screen independent factors, which were conducive to fitting a more efficient prediction model; (3) this study used training and external validation sets to evaluate the model’s discrimination ability, calibration ability, accuracy, and clinical utility, thereby comprehensively demonstrating its ability to predict liver fluke infection on the basis of laboratory indicators; and (4) this study revealed that the diagnostic ability of the combined model for liver fluke infection was superior to that of previously identified serum markers (EO# and GGT).

In general, liver flukes parasitize the biliary system, causing damage to both the digestive system and the liver. Currently, no published studies have established a connection between liver fluke infection and heart disease. However, cardiovascular disease emerged as the most common complication of liver fluke infection in our study, with coronary atherosclerotic heart disease accounting for more than 50% of cases. The underlying mechanisms for this observation require further investigation. This study revealed that significantly more male than female patients consumed raw fish while drinking alcohol, potentially because of male dietary habits [16, 17]. Previous studies have shown that infection with *C. sinensis* can affect biochemical indicators of liver function, especially ALT, aspartate aminotransferase (AST), GGT, and HDL-C, which is consistent with the results of this study [12, 18]. GGT was included in the model established in this study, which was consistent with previous studies and was considered to have predictive ability for liver fluke infection. GGT is known to be a relatively sensitive indicator of alcohol. Owing to the limitations of retrospective data collection in this study, we were unable to fully assess the alcohol consumption history of patients, which might have resulted in unknown biases. HDL-C was closely associated with blood lipid levels, while adult *C. sinensis* inhabited a high-fat and low-sugar biliary environment. The survival and activity of *C. sinensis* might be related to the high lipid environment in the biliary tract, and the elevation of lipids could increase its virulence and promote the continued parasitization of *C. sinensis* in the human body. As the end product of purine metabolism, UA has been demonstrated to significantly influence dyslipidemia regulation [19]. Elevated UA levels increase the risk of dyslipidemia, which is closely related to fatty acids formed by triglyceride metabolism [20]. Therefore, both UA and HDL-C may affect the living environment of liver flukes by regulating blood lipid levels. Fg was incorporated into the final predictive model, likely due to the high fibrinolytic activity of cathepsin L, a key component of the protein secreted by liver flukes. This enzyme effectively degrades fibrin and influences the Fg level in the bloodstream [21]. Additionally, indicators such as kidney function and electrolyte levels were included in the predictive model developed in this study. Liver fluke infection might impose a severe burden on renal health through immune complex-mediated glomerular disease, which could lead to abnormal renal function indicators [22]. Xie et al. reported that liver fluke infection is associated with renal dysfunction, revealing a significant difference in the eGFR between infected and noninfected groups; however, the underlying mechanism remains unclear [23]. In addition, the kidneys are the primary organs in healthy individuals that retain and excrete electrolytes and fluids, and kidney disease may cause an imbalance in electrolyte levels [24]. Notably, in our predictive model, *P* demonstrated a relatively high odds ratio, which may be attributed to the following reasons: (1) after infection, *C. sinensis* parasitizes the hepatobiliary ducts, which may lead to changes in the local microenvironment and indirectly affect *P* metabolism balance. Studies have shown that liver fluke infection can cause bile duct fibrosis, cholestasis, and liver cirrhosis, which may interfere with phosphate enterohepatic circulation and excretion [25]. (2) *P* may participate in immunoregulatory mechanisms following liver fluke infection. As a key component of adenosine triphosphate and nucleic acids, phosphate is involved in energy metabolism and immune cell function. Abnormal phosphate metabolism might impair the ability of the host to clear parasites [26]. Additionally, liver fluke infection activates Th2-type immune responses, while elevated phosphate levels may promote inflammatory cytokine release, thereby exacerbating bile duct inflammation [27, 28]. The generated model also included RDW and PDW indicators reflecting red blood cell and platelet morphology, which might be due to the parasite invading the human body and stimulating an immune response involving cytokine and antibody release [29]. These immune reactions might interfere with platelet generation/maturation in the bone marrow, leading to altered platelet counts and morphology. Moreover, parasites themselves might directly damage erythroid progenitor cells, causing reduced erythropoiesis or morphological changes [30].

In this study, early changes in laboratory indicators were used to develop a combined model for predicting liver fluke infection. The ROC curve was used to evaluate the model’s discriminatory performance, revealing that the training set’s AUC (0.928) was significantly greater than that of traditional single biomarkers (EO#: 0.577; GGT: 0.620). These results confirmed the necessity of multi-index combinations to overcome the limited discriminatory power of single indicators. Among similar models, Liu et al. established a combination model on the basis of radiomic features to predict liver fluke infection in patients with hepatocellular carcinoma, achieving an AUC of 0.893 [31]. Our study demonstrated superior discrimination using laboratory indicators alone, highlighting their potential for early screening. The calibration curve revealed high consistency between the model’s predicted probabilities and actual observed probabilities in the training set, with the curve fitting well and approaching the *y* = *x* line. In the confusion matrix, the training set had 85.23% overall accuracy versus 66.33% in the external verification set. This performance difference may reflect the limited sample size of the validation set, causing estimation fluctuations. Despite accuracy variations, the model maintained stable discriminative ability (AUC = 0.808) and good calibration (Hosmer–Lemeshow test *P* = 0.162) in the validation set and maintained a balance between a sensitivity of 62.5% and a specificity of 64.29%, avoiding clinical risks from single-parameter optimization. DCA quantified the model’s net benefit across threshold probabilities, guiding clinical applications. In the training set, when the threshold probability > 49.3%, our model provided greater net benefit than the “Treat All” and “Treat None” strategies did. In the validation set, the net benefit was relatively high within the threshold range of 67–84%, supporting the targeted application of this model for diagnostic assistance in high-risk populations.

This study had several limitations: (1) sample size was limited (*n* = 147), which might affect the statistical power. Further validation using large external datasets and prospective cohorts is needed. (2) Because this was a retrospective study, some laboratory test results were missing. Data imputation was performed for indicators with a missing data rate of less than 30%. (3) The results of the first laboratory examination after the patient’s hospitalization were included in this study, but it is still impossible to guarantee that the changes in these parameters were not affected by drugs. Owing to the limitations of this retrospective study, we were unable to intervene in the drug use of patients. (4) Chest X-ray and computed tomography (CT) imaging data were not collected, and the influence of imaging features on patients infected with liver fluke could not be analyzed. (5) Logistic regression, a fundamental machine learning method, was chosen for its balance between interpretability and predictive performance. However, the use of more complex artificial intelligence (AI) models (e.g., ensemble learning) may improve accuracy in future studies with larger datasets [32, 33].

## Conclusions

On the basis of routine laboratory data, we developed and validated a nomogram model for predicting early liver fluke infection in patients. The discrimination, goodness of fit, and clinical utility of the model were evaluated through ROC curves, calibration curves, and DCA. The diagnostic efficacy of the nomogram model established in this study surpassed that of previously identified serum markers (EO# and GGT). This model may serve as a valuable reference for the clinical identification of early liver fluke infection.

## Data Availability

No datasets were generated or analyzed during the current study.

## Abbreviations

WBC: White blood cell
NE#: Neutrophil count
LY#: Lymphocyte count
MO#: Monocyte count
EO#: Eosinophil count
BA#: Basophil count
NE%: Neutrophil percentage
LY%: Lymphocyte percentage
MO%: Monocyte percentage
EO%: Eosinophil percentage
BA%: Basophil percentage
RBC: Red blood cell
HGB: Hemoglobin
HCT: Hematocrit
MCV: Mean corpuscular volume
MCH: Mean corpuscular hemoglobin
MCHC: Mean corpuscular hemoglobin concentration
RDW: Red blood cell distribution width
PLT: Platelet count
PCT: Plateletcrit
MPV: Mean platelet volume
PDW: Platelet distribution width
APTT: Activated partial thromboplastin time
PT: Prothrombin time
PTA: Prothrombin time activity
TT: Thrombin time
INR: International normalized ratio
Fg: Fibrinogen
GLU: Glucose
Cr: Creatinine
UA: Uric acid
BUN: Blood urea nitrogen
eGFR: Estimated glomerular filtration rate
TP: Total protein
ALB: Albumin
GLB: Globulin
A/G: Albumin-globulin ratio
AST: Aspartate aminotransferase
ALT: Alanine aminotransferase
ALP: Alkaline phosphatase
GGT: γ-Glutamyl transpeptidase
TBIL: Total bilirubin
DBIL: Direct bilirubin
IBIL: Indirect bilirubin
CK: Creatine kinase
LDH: Lactate dehydrogenase
TG: Triglyceride
TC: Total cholesterol
HDL-C: High-density lipoprotein cholesterol
LDL-C: Low-density lipoprotein cholesterol
Na: Sodium
K: Potassium
Cl: Chloride
Ca: Calcium
P: Phosphorus
Mg: Magnesium
FT_3_: Free triiodothyronine
FT_4_: Free tetraiodothyronine
TSH: Thyrotropin
$\overline{x }\pm s$: Average ± standard deviation
*M* (*P*_25_, *P*_75_): Median (25th percentile, 75th percentile)
*B*: Regression coefficient
OR: Odds ratio
*CI*: Confidence interval
VIF: Variance inflation factor
ROC: Receiver operating characteristic
AUC: Area under curve
DCA: Decision curve analysis
TP: True-positive
TN: True-negative
FP: False-positive
FN: False-negative

## Code availability

No custom code has been used in this study.
